# Acute Fatty Liver of Pregnancy Complicated With Mild Encephalitis/Encephalopathy With a Reversible Splenial Lesion: A Case Report

**DOI:** 10.7759/cureus.49152

**Published:** 2023-11-20

**Authors:** Shota Suzuki, Ryo Higashide, Fumiko Tsubata, Masae Sakamoto, Koji Shimabukuro

**Affiliations:** 1 Department of Obstetrics and Gynecology, Tsuchiura Kyodo General Hospital, Ibaraki, JPN

**Keywords:** unconsciousness, percutaneous liver biopsy, critical care in obstetrics, acute encephalitis, mild encephalopathy/aseptic encephalitis with reversible splenial lesion of the corpus callosum, acute fatty liver of pregnancy

## Abstract

Acute fatty liver of pregnancy (AFLP) is a rare complication of pregnancy that may result in fulminant hepatic failure. A 28-year-old woman, at 36 weeks of gestation, presented to a maternal-fetal outpatient clinic with fever and headache. She was prescribed analgesics and was planned for follow-up. Two days later, she was taken back for evaluation by her husband to the previous physician again because of the subacute onset of impaired consciousness. Blood tests showed a marked elevation of liver enzymes and C-reactive protein (CRP), and the patient was transported to a tertiary hospital. A clinical diagnosis of AFLP or hemolysis, elevated liver enzymes, and low platelets (HELLP) syndrome was made, and an emergency cesarean section was performed. Unconsciousness was prolonged due to mild encephalitis/encephalopathy with a reversible splenial lesion (MERS) for three days. A liver biopsy was performed on postoperative day 11. Liver biopsy results showed large and small droplet fatty deposits, and the diagnosis of AFLP was confirmed. Thereafter, the elevated liver enzymes resolved spontaneously solely by supportive care. The patient presented with symptoms of impaired consciousness due to mild encephalitis/encephalopathy with a reversible splenial lesion (MERS), which led to a visit to a tertiary hospital and early intervention for AFLP. This case suggested that there may be similarities between the two pathologies of AFLP and MERS.

## Introduction

Acute fatty liver of pregnancy (AFLP) was first described in 1940 by Sheehan as “obstetric acute yellow atrophy” because the clinical presentation resembled fulminant hepatitis [1]. The incidence of AFLP is reported to be one to three cases per 10,000 deliveries [2]. Most cases of AFLP occur at 36-40 weeks of gestation, and the initial symptoms are often non-specific, such as vomiting, epigastric pain, and malaise, causing a delay in diagnosis [3]. As the disease progresses, jaundice appears, and the patient develops multi-organ failure, including liver and kidney failure, complicated by disseminated intravascular coagulation (DIC) and can be fatal. Treatment is rapid delivery and intensive maternal care [4]. The detailed mechanisms of pathogenesis remain unclear, and no specific treatment has been found. The association between long-chain 3-hydroxyacyl-coenzyme A dehydrogenase (LCHAD), an enzyme involved in mitochondrial fatty acid β-oxidation, -deficient fetuses, and maternal AFLP has been reported [5], but only a partial relationship has been demonstrated, and the etiology has not been elucidated.

Mild encephalitis/encephalopathy with a reversible splenial lesion (MERS) is a clinicoradiological syndrome characterized by mild neurological symptoms and good prognosis and magnetic resonance imaging (MRI) findings of reversible symmetrical lesions with high signal on diffusion-weighted imaging (DWI) and reduced apparent diffusion coefficient (ADC) values in the corpus callosum [6]. MERS is often found in children and is frequently caused by infectious diseases such as influenza viruses and rotaviruses. Convulsions and disorders of consciousness may occur, but they usually have a good prognosis, and most patients recover without sequelae solely by supportive care [7]. Its pathogenesis remains unclear, and although there are specific treatments such as steroid pulse therapy and high-dose gamma globulin therapy as well as supportive care, there is still no evidence-established treatment [7]. Here, we present a case of a 28-year-old pregnant woman who developed AFLP and MERS simultaneously with full recovery due to prompt delivery and intense supportive care. We also discuss the possible correlation between AFLP and MERS.

## Case presentation

A 28-year-old woman, with no remarkable past medical history, gravida 2, para 1 at 36 week’s gestation presented to a maternal-fetal outpatient clinic with a fever and headache. She only consumed alcohol occasionally. Her body mass index (BMI) before pregnancy was 27. She was prescribed analgesics and was planned for follow-up. Two days later, she was taken back for evaluation by her husband to the previous physician again because of the subacute onset of impaired consciousness. Blood tests showed a marked elevation of liver enzymes and C-reactive protein (CRP), and she was transported to a tertiary hospital.

Her consciousness was Glasgow coma scale (GCS) E4V4M5, body temperature was 40.6℃, pulse rate was 147 /min, respiratory rate was 24 /min, oxygen saturation was 99% in room air, and blood pressure was 116/91 mmHg. The abdomen was nontender. Abdominal ultrasound showed increased liver brightness. There were no findings to suggest non-reassuring fetal status (NRFS) by cardiotocogram.

A blood test showed a white blood cell count of 8,830/μL, platelet count of 133,000/μL, hemoglobin concentration of 10.7 g/dL, and serum concentration of urea nitrogen of 8 mg/dL, creatinine of 0.51 mg/dL, uric acid of 8.0 mg/dL, ammonia of 17 μg/dL, amylase of 60 U/L, aspartate transaminase (AST) of 566 U/L, alanine transaminase (ALT) of 345 U/L, total bilirubin of 1.4 mg/dL, gamma-glutamyl transpeptidase (γ-GTP) of 83 U/L, CRP of 26.27 mg/dL, procalcitonin of 4.65 ng/mL, and glucose of 110 mg/dL. Coagulogram revealed a prothrombin time of 11.9 seconds with an international normalized ratio (INR) of 0.99, activated partial thromboplastin time of 39.6 seconds, fibrinogen of 904 mg/dL, and fibrin degradation products (FDP) of 93.9 μg/mL (Table 1). An antigen test for COVID-19 and influenza were both negative. A computed tomography (CT) scan of the head showed no evidence of intracranial hemorrhage.

**Table 1:** Blood test results of the patient.

Parameters	Results	Reference range
Hemoglobin	10.7 g/dL	11.6-14.8 g/dL
White blood cell count (WBC)	8830 /μL	3200-8600 /μL
Segmented cell (Seg)	79.5%	
Lymphocytes (Lymph)	14.0%	
Platelets	13.3 ×10⁴/μL	16.0-35.3 ×10⁴/μL
Fibrinogen	904 mg/dL	150-400 mg/dL
Prothrombin time (PT)	11.9 seconds	9.0-13.0 seconds
Prothrombin time-international normalized ratio (PT-INR)	0.99	0.80-1.20
Activated partial thromboplastin time (APTT)	39.6 seconds	23-40 seconds
Antithrombin III (ATIII)	60%	80-140 %
Fibrin degradation products (FDP)	93.9 μg/mL	<5 μg/mL
Urea nitrogen (UN)	8 mg/dL	8-20 mg/dL
Creatinine	0.51 mg/dL	0.46-0.79 mg/dL
Uric acid (UA)	8.0 mg/dL	2.6-5.5 mg/dL
Sodium (Na)	133 mmol/L	138-145 mmol/L
Potassium (K)	3.5 mmol/L	3.6-4.8 mmol/L
Chlorine (Cl)	100 mmol/L	101-108 mmol/L
Calcium (Ca)	7.9 mg/dL	8.8-10.1 mg/dL
Magnesium (Mg)	1.7 mg/dL	1.8-2.4 mg/dL
Ammonia (NH3)	17 μg/dL	12-66 μg/dL
Amylase	60 U/L	44-132 U/L
Asparate aminotransferase (AST)	566 U/L	13-30 U/L
Alanine aminotransferase (ALT)	345 U/L	7-23 U/L
Total bilirubin	1.4 mg/dL	0.4-1.5 mg/dL
Gamma-glutamyl transpeptidase (γ-GTP)	83 U/L	9-32 U/L
Lactate dehydrogenase (LD)	1238 U/L	124-222 U/L
Creatine kinase (CK)	160 U/L	41-153 U/L
Triglycerides (TG)	459 mg/dL	30-117 mg/dL
C-reactive protein (CRP)	26.27 mg/dL	≦0.14 mg/dL
Procalcitonin	4.65 ng/mL	≦0.05 ng/mL
Glucose	110 mg/dL	80-112 mg/dL

**Figure 1:**
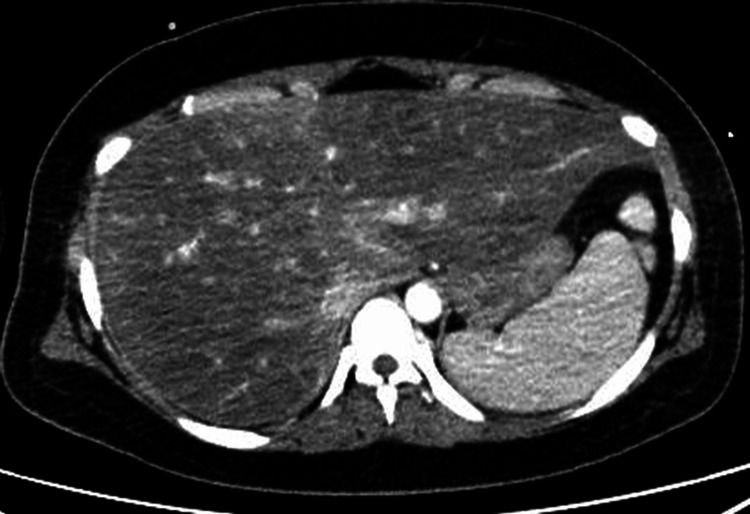
Axial abdominal contrast-enhanced CT image taken postoperatively shows marked fatty liver and hepatomegaly.

AFLP or hemolysis, elevated liver enzymes, and low platelets (HELLP) syndrome was suspected due to markedly elevated liver enzymes, and an emergency cesarean section was performed under general anesthesia on the same day. A live male baby was delivered with a birth weight of 2,602 g and an Apgar score of 2 at one minute and 8 at five minutes after birth and did not require admission to the neonatal intensive care unit. Maternal impaired consciousness continued postoperatively, but blood tests, a cerebrospinal fluid (CSF) analysis, and a contrast-enhanced CT scan did not reveal any cause of consciousness disturbance. An electroencephalography was performed, and there were no signs that could cause unconsciousness, and non-convulsive status epilepticus was ruled out. CT images showed marked fatty liver and hepatomegaly (Figure 1).

**Figure 2:**
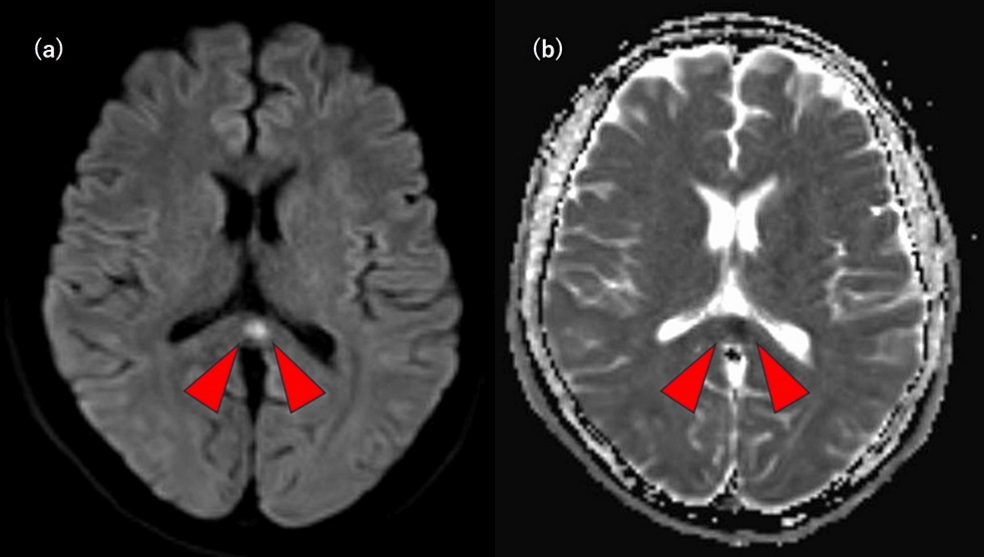
A head MRI image of postoperative day one. (a) DWI image shows symmetrical lesions with high signal in the corpus callosum (red arrowhead). (b) ADC map image shows symmetrical lesions with reduced ADC values in the corpus callosum (red arrowhead).

An MRI scan of the head was performed on postoperative day one, and she was diagnosed with MERS (Figure 2).

**Figure 3:**
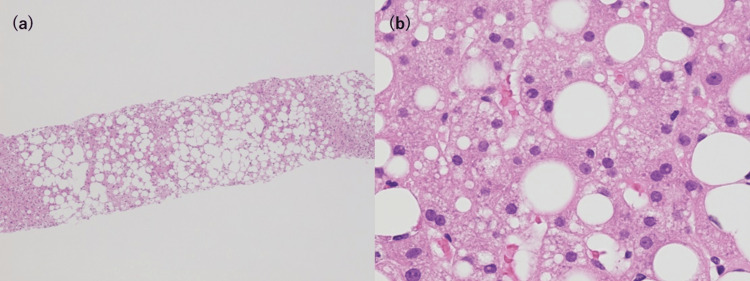
Pathological findings of liver biopsy on operative day 11. (a) ×40: The majority of liver tissue is fatty tissue. (b) ×400: Small and large droplet fatty deposits are present.

Thereafter, the patient's conscious state improved on postoperative day three solely by supportive care. At about the same time, fever resolution was also achieved only by providing antipyretics. However, liver enzyme elevations were persistent, and screening tests for autoimmune and viral hepatitis were negative, so a liver biopsy was performed on postoperative day 11. Liver biopsy results showed large and small droplet fatty deposits (Figure 3).

Although pathology suggested that the patient may have originally had a fatty liver, the diagnosis of AFLP was made in conjunction with the clinical course.

On postoperative day 12, the patient was discharged from the hospital in good general condition. Thereafter, the elevated liver enzymes resolved spontaneously. No congenital metabolic abnormalities or other laboratory abnormalities were found in the baby.

## Discussion

AFLP, reported by Sheehan in 1940, is a fatal disease for both the mother and the fetus that mainly develops in the third trimester of pregnancy or postpartum period with nonspecific symptoms, such as epigastric pain, headache, vomiting, and fatigue, and, in severe cases, with jaundice, DIC, renal failure, and hepatic encephalopathy [4]. Its occurrence is rare, the prevalence is estimated to be one to three cases per 10,000 deliveries [2]. AFLP is more common in primiparous women, male pregnancies, and multiple pregnancies and is complicated by hypertensive disorders of pregnancy in about half of the cases [8]. The cause of AFLP is not yet clear, but an association with genetic abnormalities in LCHAD, which is involved in mitochondrial fatty acid beta-oxidation, has been reported, suggesting that fatty acid beta-oxidation and impaired lipoprotein synthesis and transfer may be involved in its pathogenesis [9]. Laboratory findings include elevated AST, ALT, ALP, ammonia, direct bilirubin, increased white blood cell counts, renal dysfunction, and findings of DIC. Treatment consists of rapid delivery and intensive maternal care [10]. On clinical and laboratory examination, it is difficult to differentiate AFLP from viral fulminant hepatitis and HELLP syndrome. AFLP shows characteristic pathological findings such as lobulocentric microdroplet-like fatty deposits, and, unlike fulminant hepatitis, there are few inflammatory changes such as parenchymal necrosis or cellular infiltration of the portal venous region [11,12]. However, as AFLP is often associated with DIC at onset, a liver biopsy cannot be performed, and a definitive diagnosis is often not possible in practice. Although there is no consensus on the diagnostic criteria for clinical AFLP, the Swansea criteria are often used as diagnostic criteria [13]. In our case, a liver biopsy was performed after the coagulation abnormalities had improved, and by including the pathology results, this case matched the diagnostic criteria for AFLP.

MERS is a group of symmetrical lesions with MRI findings of high signal on DWI and reduced ADC values in the cerebral corpus callosum, associated with mild neurological symptoms and a good prognosis. It is caused by a variety of causes, including infections such as influenza viruses, drugs, and metabolic abnormalities, and is most common in children. The detailed pathogenesis of MERS is not yet clear, but it has been reported that it may be caused by transient local infiltration of inflammatory cells, many of which occur secondary to viral infections. Shi et al. reported that several inflammation cytokines can stimulate the hypothalamus and pituitary gland and induce vasopressin release, causing hyponatremia and resulting in cerebral edema. They described that the high myelin density in the cerebral corpus callosum may be the cause of selective lesions [7]. Though convulsion and disorder of consciousness may occur, most patients recover without sequelae solely by supportive care [6]. In the present case, the cause of unconsciousness was determined to be MERS, as the MRI showed findings of MERS, and no other cause was found.

This case study suggested that there may be similarities between the two pathologies of AFLP and MERS. Previous case series of AFLP reported that, among 17 patients diagnosed with AFLP, six were reported to have developed the disease secondary to inflammatory gastrointestinal diseases, five of whom had inflammatory bowel disease and one had influenza hepatitis [4]. Another report of AFLP demonstrated the possible mechanism of AFLP by the release of cytokine by Kupffer cells during infection [14]. In the present case, fever and headache preceded AFLP, suggesting the inflammation of the body may have triggered AFLP. The markedly high CRP and procalcitonin values suggest strong systemic inflammation and possibly hypercytokinemia. Among various inflammatory cytokines, TNF-α is a common cytokine and has the ability to damage intracellular mitochondria and induce necrosis and apoptosis [15,16]. Peroxisome proliferator-activated receptor (PPAR) is a receptor that induces peroxisome proliferation in cells and are nuclear receptor-type transcription factor that regulates the expression of a group of genes closely related to sugar and lipid metabolism and cell differentiation. There are three subtypes, α, β, and γ. PPARα is mainly distributed in the liver, kidney, heart, and skeletal muscle and plays an important role in fatty acid metabolism [17]. Cobbina et al. reported that TNF-α has been implicated in the alterations of nuclear factors such as PPAR-α in non-alcoholic fatty liver disease (NAFLD) [18]. PPARα has been reported to have β-oxidative and anti-TNF-α effects [19,20]. Therefore, this case study suggested that inflammation might be the trigger for both AFLP and MERS.

The liver biopsy, in this case, showed a severe fatty liver, with not only small droplet fatty deposits but also large droplet fatty deposits. There were no pathological findings such as autoimmune or fulminant hepatitis and no positive findings in serological screening tests for hepatitis. Although the possibility that the patient originally had NAFLD cannot be ruled out on the basis of pathological findings alone, the diagnosis of AFLP was made in conjunction with the clinical course of the disease.

In this case, the episode of impaired consciousness allowed an early diagnosis of AFLP, resulting in prompt cesarean section before the appearance of severe symptoms of AFLP. AFLP is often detected in a more advanced state and is a highly lethal disease for both mother and fetus [3]. In this case, the patient presented with symptoms of impaired consciousness due to MERS, which led to a visit to a tertiary hospital and early intervention with AFLP. Further research and more cases of AFLP may reveal a more detailed pathogenesis of AFLP and MERS and their similarities.

## Conclusions

The detailed pathogenesis of AFLP remains unclear, and diagnostic criteria and treatment have not yet been established. In this case, inflammation may have triggered AFLP and MERS. Further research and more cases of AFLP may reveal a more detailed pathogenesis of AFLP and MERS and their similarities.
